# Roles of HDAC3-orchestrated circadian clock gene oscillations in diabetic rats following myocardial ischaemia/reperfusion injury

**DOI:** 10.1038/s41419-020-03295-y

**Published:** 2021-01-07

**Authors:** Zhen Qiu, Hao Ming, Shaoqing Lei, Bin Zhou, Bo Zhao, Yanli Yu, Rui Xue, Zhongyuan Xia

**Affiliations:** 1 Department of Anesthesiology, Renmin Hospital of Wuhan University, 430060 Wuhan, Hubei China; 2 Department of Anesthesiology, Renmin Hospital, Hubei University of Medicine, 442000 Shiyan, Hubei China

**Keywords:** Transcriptional regulatory elements, Myocardial infarction, Type 1 diabetes

## Abstract

The circadian clock is closely related to the development of diabetes mellitus and cardiovascular disease, and disruption of the circadian clock exacerbates myocardial ischaemia/reperfusion injury (MI/RI). HDAC3 is a key component of the circadian negative feedback loop that controls the expression pattern of the circadian nuclear receptor Rev-erbα to maintain the stability of circadian genes such as BMAL1. However, the mechanism by which the HDAC3-orchestrated Rev-erbα/BMAL1 pathway increases MI/RI in diabetes and its relationship with mitophagy have yet to be elucidated. Here, we observed that the clock genes Rev-erbα, BMAL1, and C/EBPβ oscillations were altered in the hearts of rats with streptozotocin (STZ)-induced diabetes, with upregulated HDAC3 expression. Oscillations of Rev-erbα and BMAL1 were rapidly attenuated in diabetic MI/R hearts versus non-diabetic I/RI hearts, in accordance with impaired and rhythm-disordered circadian-dependent mitophagy that increased injury. Genetic knockdown of HDAC3 significantly attenuated diabetic MI/RI by mediating the Rev-erbα/BMAL1 circadian pathway to recover mitophagy. Primary cardiomyocytes with or without HDAC3 siRNA and Rev-erbα siRNA were exposed to hypoxia/reoxygenation (H/R) in vitro. The expression of HDAC3 and Rev-erbα in cardiomyocytes was increased under high-glucose conditions compared with low-glucose conditions, with decreased BMAL1 expression and mitophagy levels. After H/R stimulation, high glucose aggravated H/R injury, with upregulated HDAC3 and Rev-erbα expression and decreased BMAL1 and mitophagy levels. HDAC3 and Rev-erbα siRNA can alleviate high glucose-induced and H/R-induced injury by upregulating BMAL1 to increase mitophagy. Collectively, these findings suggest that disruption of HDAC3-mediated circadian gene expression oscillations induces mitophagy dysfunction, aggravating diabetic MI/RI. Cardiac-specific HDAC3 knockdown could alleviate diabetic MI/RI by regulating the Rev-erbα/BMAL1 pathway to restore the activation of mitophagy.

## Introduction

Diabetes mellitus (DM) is a major public health challenge with a continuously increasing prevalence, currently estimated 1/8 population of worldwide; it is predicted to be 640 million diabetic patients worldwide, in 2040^1^. Ischaemic heart disease is the main cardiovascular complication of diabetes and the leading cause of death^2^. Increasing evidences, including our previous studies, has shown not only that the incidence of myocardial ischaemia in diabetes patients is obviously higher than that in non-diabetic individuals but also that these patients are more susceptible to myocardial ischaemia/reperfusion injury (MI/RI), with larger infarct sizes and higher new congestive heart failure rates^3–6^. However, the specific mechanisms by which diabetes aggravates MI/RI are not clear. Therefore, further clarification of the pathological mechanism of diabetic MI/RI and more effective prevention measures to improve prognosis are important issues to be resolved in this field.

The circadian clock is an important endogenous regulatory mechanism that exists in a wide range of living organisms. Circadian clock gene oscillations play an important regulatory role in diabetes, cardiovascular physiology, and pathophysiology^7,8^. In the type 1 diabetes rat model, the expression of core clock genes (Rev-erbα, BMAL1) and output genes (dbp, hlf) showed a significant phase shift, which was manifested by advancement of the rhythm^9^. Moreover, recent research has indicated that the intrinsic circadian rhythm of cardiomyocytes may contribute to the time-of-day dependence of cardiovascular physiology. The onset of myocardial infarction also exhibits a marked circadian rhythm in humans, with a higher incidence in the early hours of the morning than in the evening^10^. Perioperative myocardial injury in patients undergoing aortic valve replacement is closely transcriptionally regulated by the circadian clock^11^. Ronald Reiter et al. reported that myocardial infarct size and left ventricular function following STEMI show a circadian dependence on the time of day of the onset of ischaemia^12^.

HDAC3 is an important mediator of glucose metabolism and fat metabolism and plays a key role in the circadian rhythm negative feedback loop; the activation of HDAC3 is essential for maintaining the normal circadian rhythm^13^. HDAC3 is required for Rev-erbα activity. Rev-erbα gene regulates the circadian rhythm by binding sites in BMAL1 gene promoter and the expression of BMAL1 is negatively correlated with the mRNA level of Rev-erbα^14,15^. On the other hand, HDAC3 activity contributes to ischaemic cardiac damage^16^. Injection of HDAC3 inhibitor before or during reperfusion can reduce infarct size and maintain cardiac systolic function^17^. Circadian clock gene oscillations (i.e., Rev-erbα and BMAL1) were rapidly attenuated in the I/R group versus the non-ischaemic region and sham group^18^. The amplitude of Rev-erbα and BMAL1 oscillation in the ischaemic region was rapidly attenuated, and the synchrony of the circadian clock in the ischaemic and non-ischaemic regions exacerbated MI/RI^19,20^. Knockout or inhibition of Rev-erbα gene reduced myocardial damage in a sleep–awake model of MI/R in mice^11^.

Mitophagy is a key endogenous protective mechanism that maintains complete mitochondrial network function, oxidative balance, and cell survival^21^. Recent studies have indicated that mitophagy is rhythmically related to clock gene oscillation, whose cyclic induction may provide a novel link between the clock and metabolism^22^. Further studies have shown that the transcription factor CCAAT/enhanced binding protein beta (C/EBPβ) is a clock gene that acts as an important transcription factor regulating mitophagy activity; maintenance of mitophagy rhythm depends on the regulation of the core clock gene BMAL1 and is associated with the activation of C/EBPβ^23,24^. In diabetic state, hyperglycaemia-induced and hyperlipidaemia-induced oxidative stress increases mitophagy dysfunction^25^. Mitophagy is more severe in type 1 diabetes after MI/R insult^6^. However, the underlying mechanisms by which circadian mitophagy rhythm is regulated by the clock gene oscillation loop signalling pathway and whether mitophagy rhythm disorder is an important mechanism of increased MI/RI vulnerability in the diabetic state have not yet been clarified.

Therefore, in this study, we explored the mechanism by which the HDAC3-mediated Rev-erbα/BMAL1 pathway increases MI/RI vulnerability in diabetes and its relationship with mitophagy rhythm.

## Materials and methods

### Experimental animals

SPF healthy male Sprague-Dawley (SD) rats weighing 200–220 g, 6–8 weeks of age, were purchased from Beijing Vital River Laboratory Animal Technology Co., Ltd. Rats were kept at the Animal Experimental Center of Renmin Hospital of Wuhan University under constant temperature and humidity with a strick 12-h light/dark cycle regime and free access to water and food. The light time is 7 a.m.–7 p.m. (zeitgeber time (ZT) 0–ZT12), and the dark time is 7 p.m.–7 a.m. (ZT12–ZT24). Experimental protocols were implemented after being reviewed and approved by the Laboratory Animal Welfare & Ethics Committee (IACUC) of Renmin Hospital of Wuhan University. All animal procedures have conformed to the guidelines from Directive 2010/63/EU of the European Parliament on the protection of animals used for scientific purposes or the NIH Guide for the Care and Use of Laboratory Animals.

### Establishment of type 1 diabetic rat model

After fasted 12 h, rats were administrated by intraperitoneal injection 60 mg/kg streptozotocin (STZ) (Sigma, USA) to establish diabetes model as described previously^4–6^. The fasting blood glucose (after fasting for 6 h) was measured after 72 h. The hallmarks of successful establishment of diabetes model are blood glucose levels ≥16.7 mmol/L with increased consumption of food and water and increased urination of rats. After that, all rats are continuously raised for 8 weeks. With the premise of statistical significance and repeatability, we use the smallest sample size for all animal experiments. There are six successful modelling samples for each related indicators in each group of diabetic rats or non-diabetic rats, respectively.

### AAV9 infection

To examine the effects of HDAC3 gene knockdown in MI/R‐stimulated diabetic rats, we used recombinant adeno‐associated virus serotype 9 (AAV9) vectors which carry a CMV promoter with GFP reporter (HBAAV9-HDAC3 shRNA1-GFP) or HBAAV9-GFP NC which were produced by Hanbio Biotechnology Co., to knock down HDAC3 gene expression or as control. AAV-HDAC3 was given via tail vein injection at a dose of 2 × 10^12^ vg/kg 3 weeks before I/R insult.

### Experimental model of MI/RI

**Fig. 1:**
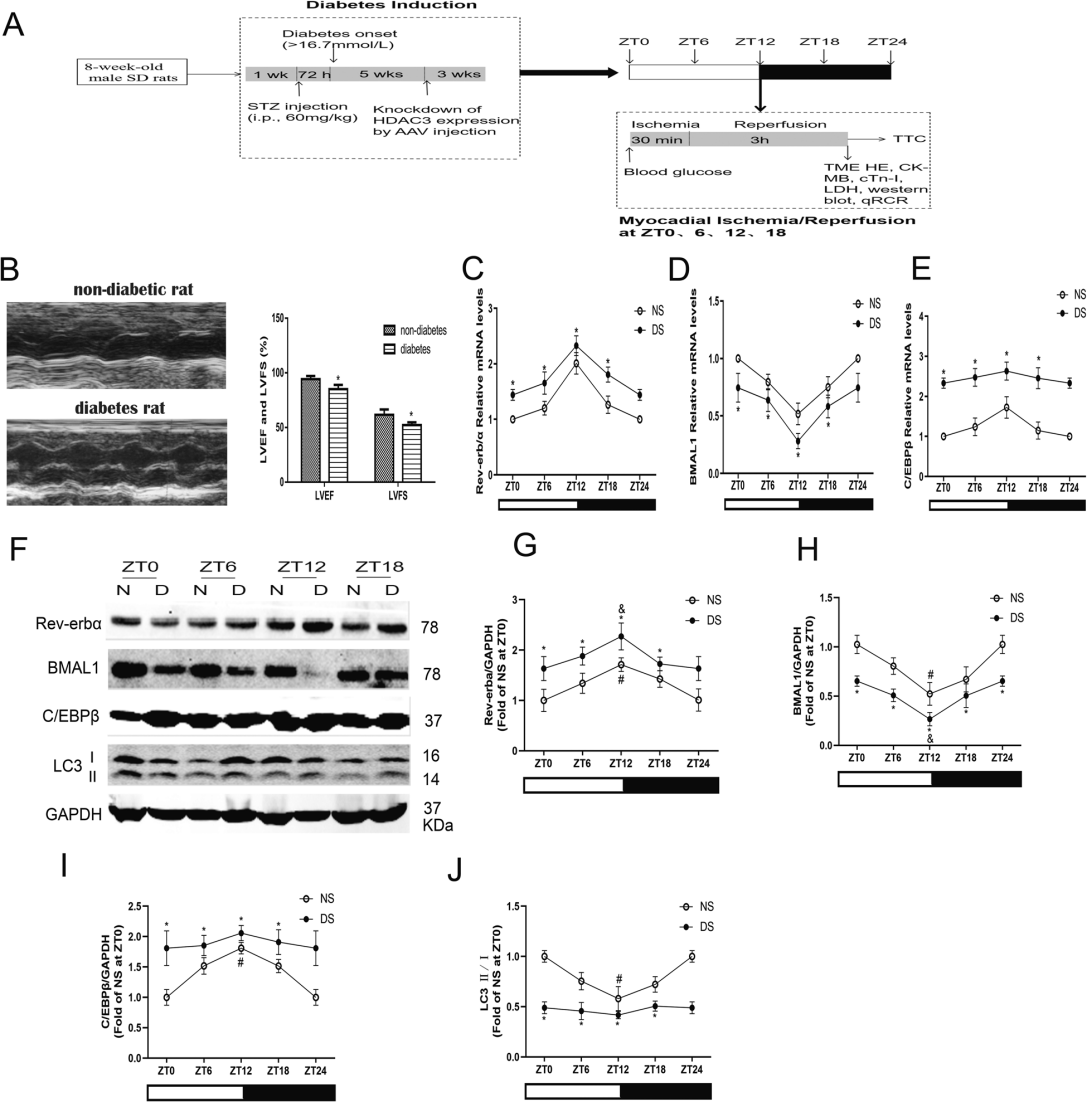
Circadian clock gene rhythmicity was altered with attenuated autophagy in the hearts from STZ-induced diabetic rats. **A** Schematic diagram of experimental programme. **B** Cardiac function was assessed by echocardiography in non-diabetic and diabetic rats. Scale bar: 2 mm. **C**–**E** Expression levels of Rev-erbα (**C**), BMAL1 (**D**), and C/EBPβ (**E**) mRNA were measured by qPCR over time after reperfusion. **F**–**J** The protein levels of Rev-erbα (**G**), BMAL1 (**H**), C/EBPβ (**I**) and LC3 II/I (**J**) were detected by western blotting in the myocardial tissues of non-diabetes and diabetes. *n* = 6 per group. ^*^P < 0.05 versus NS within ZT; ^#^*P* < 0.05 versus ZT0 within NS; ^&^*P* < 0.05 versus ZT0 within DS.

The model of MI/RI was established as previously described^4–6^. Briefly, after given 1% pentobarbital sodium formulated with normal saline by intraperitoneal injection at a dose of 60 mg/kg to anaesthetize rats, the rats were received mechanical ventilation after endotracheal intubation with ECG monitoring. At the fourth intercostal space of left subclavian midline, we opened the chest of rats to expose the heart; then the left descending coronary artery (LAD) was occluded for 30 min followed by reperfusion for 2 h. Sham control group rats underwent same operation without LAD ligation. The non-diabetes group (N) and the diabetes group (D) were divided into sham (S) (*n* = 6 each group) operation group and ischaemia/reperfusion (I/R) (*n* = 12 each group) group at ZT0, 6, 12, and 18, respectively, according to the random number table method (Fig. 1A).

### Cardiac functional assessment

Cardiac function was monitored by animal ultrasound system with rat ultrasound by measuring the left ventricular EF and FS% recorded on a polygraph (RM-6240C; Chengdu Instrument Co., Ltd, China) when rats were anaesthetized by 40 mg/kg pentobarbital sodium. The measurements of two-dimensional and M-mode echocardiographic were analysed with a GE vivid 7 high-resolution in vivo-imaging system (VisualSonics, Toronto, ON, Canada).

### Infarct size measurement

At the end of reperfusion, six rats were taken from each group to detect myocardial infarct size by ligating LAD again with 0.3% evans blue dye (Sigma, USA) with 1% 2,3,5-triphenyltetrazolium chloride (TTC) (Sigma, USA) staining. Briefly, 3% Evans blue stain was slowly injected through the femoral vein until a clearly distinguish between the blue stained and non-blue stained areas of the myocardium, and then the heart was quickly obtained. After being frozen at −20 °C for half an hour, it was cut into 2 mm thick heart slices into 4–5 slices. Slices were incubated with 1% TTC solution at 37 °C. Then 4% paraformaldehyde was used to fix slices for 15–20 min. The area of red, white, and blue in myocardial tissue were detected with a scanner (Epson, v30, Japan), and myocardial infarct size were analysed by an image analysis system software Image-ProPlus as described previously^4–6^.

### Measurement of serum troponin-I

Serum troponin-I was used to evaluate myocardial injury. After reperfusion, we used assay kit (Jiancheng, Nanjing, China) to analyse the level of serum troponin-I by collecting arterial blood samples through the carotid artery.

### Transmission electron microscopy (TEM)

After reperfusion, 1 mm^3^ tissue of the left ventricle in hearts isolated from rats were collected and fixed within 2.5% glutaraldehyde for 24 h. The tissue samples were washed, fixed, dehydrated, embedded, and cured with buffer solution, then were cut into ultrathin sections by an ultra-thin microtome. The TEM micrographs of ultrathin sections were detected by TEM (Tecnai G^2^ 20 TWIN, USA) under the guidance of professional teachers of the Core Facility of Wuhan Institute of Virology.

### Histological examination (HE)

After reperfusion, tissues from the heart apical region were isolated and rinsed with phosphate buffered saline and then fixed with 4% buffered paraformaldehyde embedded in paraffin. HE staining and the images of cross sections from the heart were obtained by upright Metallurgical Microscope (Canon, Tokyo, Japan).

### In vitro experimental protocol and hypoxia/reoxygenation model

Primary neonatal rat cardiomyocytes were obtained from newborn SD suckling rats at 1–3 days. Briefly, newly born SD rats were disinfected with 75% alcohol, then chest was opened and the heart was harvested. The heart tissue fragments were digested in ADS buffer solution containing 1% collagenase from clostridium histolyticum (Sigma, USA) and 0.75% pancreatin from porcine pancreas (Sigma, USA) for six times, 20 min each time at 37 °C, 70 rpm in constant temperature shaker. Cell suspension was then centrifuged for 3 min at 2200 rpm, and the supernatant was discarded. Resuspend the obtained cell pellet into the top of the percoll gradient, centrifuge at 3000 rpm for 30 min at room temperature. Collect the cardiac cells and then was centrifuged at 2200 rpm for 3 min. Then the cells were cultured in 6-well and 96-wells plates for experiments under low glucose (LG) (5.5 mM glucose concentration) DMEM (Gibco, USA) containing 10% foetal bovine serum (Gibco, USA) and 1% penicillin and 1% streptomycin in a cell culture incubator at 37 °C within 5% CO_2_. HDAC3 siRNA and Rev-erbα siRNA (Ribobio, China) transfection were performed by using lipofectamineTM 2000 (Invitrogen, USA). After 24 h post-transfection, the cells were exposed to high glucose (HG) DMEM at a concentration of 30 mM glucose for 24 h treated at non-toxic concentrations. Subsequently, neonatal cardiomyocytes were subjected to hypoxic conditions (0.9% O_2_/94.1% N_2_/5% CO_2_) for 6 h, followed 2 h normal condition for reoxygenation.

### Cell viability assay

CCK-8 assay kit (Jiancheng, Nanjing, China) was used to measure cell viability. After stimulation, the cultured cells in 96-well plates were given 10 μl CCK-8 reagent for each well and then incubated for 3 h in darkness. Perkin Elmer Microplate reader (PerkinElmer Victor 1420, USA) was used to analyse the absorbance at 450 nm.

### Mitochondrial ROS measurement

MitoTracker Red CMXRos assays (YEASEN, China) was used to measure the mitochondrial ROS production. 500 nM MitoTracker Red CMXRos working liquid was added to the cells and incubated at 37 °C for 30 min in darkness. After that, cold PBS was used to wash the cells twice. The fluorescence intensity of mitochondrial ROS was recorded by using fluorescence microscopy (Olympus IX51, Japan).

### Assessment of mitochondrial membrane potential (MMP)

MMP was detected by JC-1 (Beyotime, China) staining according to manufacturer’s instructions. Briefly, at the end of experimental stimulation, cells were incubated with JC-1 dye working liquid at 37 °C for 20 min in darkness. Subsequently, JC-1 buffer was used to wash the cells twice. Images of cells were obtained immediately and analysed by using a fluorescence microscope (Olympus IX51, Japan). When the MMP is high, JC-1 aggregates into the matrix of the mitochondria to form a polymer (J-aggregates) which can produce red fluorescence. When the MMP is low, JC-1 cannot aggregate mitochondria in the matrix and JC-1 is a monomer at this time, and then green fluorescence can be produced.

### Measurement of autophagic flux

To measure the autophagic flux of cardiomyocytes, we used the tandem fluorescent mRFP-GFP-LC3 adenovirus (MOI = 100) to transfect cultured cells. GFP and mRFP in mRFP-GFP-LC3 adenovirus were used to label and track LC3. 24 h after adenoviral transfection, cells were washed with PBS, fixed with 4% paraformaldehyde, mounted with a reagent containing DAPI (Sigma, USA). The expression of GFP and mRFP was detected with Olympus FV1200 laser scanning confocal microscope (Olympus, Japan). Attenuation of GFP indicate that lysosomes and autophagosomes fuse to form autophagosomes, which red fluorescence can only be detected at this time. Yellow (merge of GFP signal and RFP signal) puncta represented early autophagosomes, while red (RFP signal alone) puncta indicate late autolysosomes. The autophagic flux was evaluated by counting the spots of different colours.

### Mitophagosome formation determination

After H/R stimulation, MitoTracker Green (MTG) and LysoTracker Red (LTR) were used to stain the cardiomyocytes, followed by confocal imaging of mitochondria, lysosomes, and colocalization of both markers is an indicator of mitophagosome formation. Cardiomyocytes were incubated with 300 nM MTG for 30 min, followed by 150 nM LTR for 45 min at 37 °C in darkness. Fluorescent images were detected with Olympus FV1200 laser scanning confocal microscope (Olympus, Japan). Quantitative analysis of MitoTracker-LysoTracker colocalization was presented by the merged spots.

### Gene expression

Following the manufacturer’s instructions, total RNA was extracted from myocardium and cardiomyocytes by trizol reagent (Invitrogen, USA), then reversely transcribed 1 μg total RNA into cDNA by using a reverse transcription kit (Takara, China). The mRNA levels of HDAC3, Rev-erbα, BMAL1, C/EBPβ were performed by quantitative RT-PCR using Bio-Rad CFX Connect Real-Time PCR Detection System (Bio-Rad, USA) with the following primers: HDAC3 (forward): 5′-ACCGTGGCGTATTTCTACGAC-3′; HDAC3 (reverse): 5′-CCTGGTAAGGCTTGAAGACGA-3′; Rev-erbα (forward): 5′-AAGGTTGTCCCACATACTTCCC-3′; Rev-erbα (reverse): 5′- CAGTAGCACCATGCCGTTAAG-3′; BMAL1 (forward): 5′-ACACCTAATTCTCAGGGCAGC-3′; BMAL1 (reverse): 5′-GAAGTCCAGTCTTCGCATCG-3′; C/EBPβ (forward): 5′-CGGTGGACAAGCTGAGCGACGAGTA-3′; C/EBPβ (reverse): 5′- GTTCCGCAGCGTGCTGAGCTCTC-3′; GAPDH (forward): 5′- CGCTAACATCAAATGGGGTG-3′; GAPDH (reverse): 5′-TTGCTGACAATCTTGAGGGAG-3′. The levels of mRNA were normalized relative to GAPDH. The expression of genes was analysed by using the 2^−ΔΔCT^ method.

### Western blotting

Western blot analysis was performed as described previously^20^ using antibodies against HDAC3 (1:1000, CST, #3949), Rev-erbα (1:1000, CST, #13418), BMAL1 (1:1000, CST, #14020), C/EBPβ (1:1000, abcam, ab32358), BNIP3 (1:1000, CST, #3769), Atg4b (1:1000, CST, #13507), p62 (1:1000, CST, #23214), LC3B (1:1000, CST, #3868), and GAPDH (1:1000, CST, #5174). Myocardial proteins were lysed in ice-cold radio immunoprecipitation assay buffer, and then centrifuged at 12,000 rpm at 4 °C for 15 min to collect supernatants. Protein lysates were loaded into an 5% to 10–12% SDS–PAGE gel and transferred to polyvinylidene difluoride (PVDF) membrane, and then incubated with specific primary antibodies at 4 °C overnight. Subsequently incubated with fluorescent secondary antibody for 1 h at room temperature. The protein bands were obtained by using odyssey colour infrared laser scan-imaging instrument (Li-Cor, USA).

### Statistical analysis

All data are expressed as the mean ± standard deviation. GraphPad Prism version 8.0 (GraphPad Software, USA) was used to statistical software analysis. Differences among experimental groups were analysed by ANOVA followed by post-assay/test method by Bonferroni correction for post hoc *t*-test. *P* values < 0.05 were considered to be statistically significant. All data analysis were performed by observers blinded to the experimental groups.

## Results

### Circadian clock gene rhythmicity was altered with attenuated autophagy in the hearts from STZ-induced diabetic rats

After STZ injection for 8 weeks, the STZ-induced diabetic rats showed decreased body weight and increased plasma glucose level as compared to that of non-diabetic rats (Supplementary Table 1). Echocardiography showed a decreased left ventricular ejection fraction (LVEF) and left ventricular fractional shortening (LVFS) in diabetic rats compared with non-diabetic rats (Fig. 1B). We also examined the diurnal oscillations of Rev-erbα, BMAL1 and C/EBPβ mRNA and protein levels in non-diabetic and diabetic rats at the beginning of the light phase (ZT0), the middle of the light phase (ZT6), the beginning of the dark phase (ZT12) and the middle of the dark phase (ZT18). The mRNAs encoding and protein expression of Rev-erbα, and BMAL1 had a circadian expression pattern in hearts isolated from both non-diabetic and diabetic rats (Fig. 1C–F). However, the mRNA and protein expression of Rev-erbα in diabetic hearts was much higher than that in non-diabetic hearts with lower amplitude (Fig. 1C, G). The diurnal oscillations of the clock gene BMAL1 were significantly attenuated in diabetic hearts at lower levels (Fig. 1D, H). Additionally, upregulated C/EBPβ (Fig. 1E, I) and downregulated LC3 II/I (Fig. 1J) were observed in diabetic hearts without obvious circadian than that in non-diabetic hearts.

### The circadian dependence of MI/RI in diabetic rats was attenuated, which was associated with disordered rhythm of mitophagy

To investigate the relationship between time-of-day-dependent oscillations in MI/R tolerance and the circadian rhythm, diabetic and age-matched non-diabetic rats were subjected to MI/R stimulation at ZT0, ZT6, ZT12, and ZT18. We observed diurnal variations of infarct size in non-diabetic rats after MI/RI (Fig. 1A). The infarct size of diabetic hearts was significantly increased compared that of with non-diabetic hearts, and there was no significant difference among these time points. The time-of-day dependence of infarct size in non-diabetic patients was accompanied with diurnal variations in cTn-I (Fig. 1B). The level of cTn-I was increased rapidly with attenuation/abolition of diurnal variations in diabetes compared with non-diabetes at different time points after I/R insult, and the peak of cTn-I level after ischaemic insult occurred at ZT12.

**Fig. 2:**
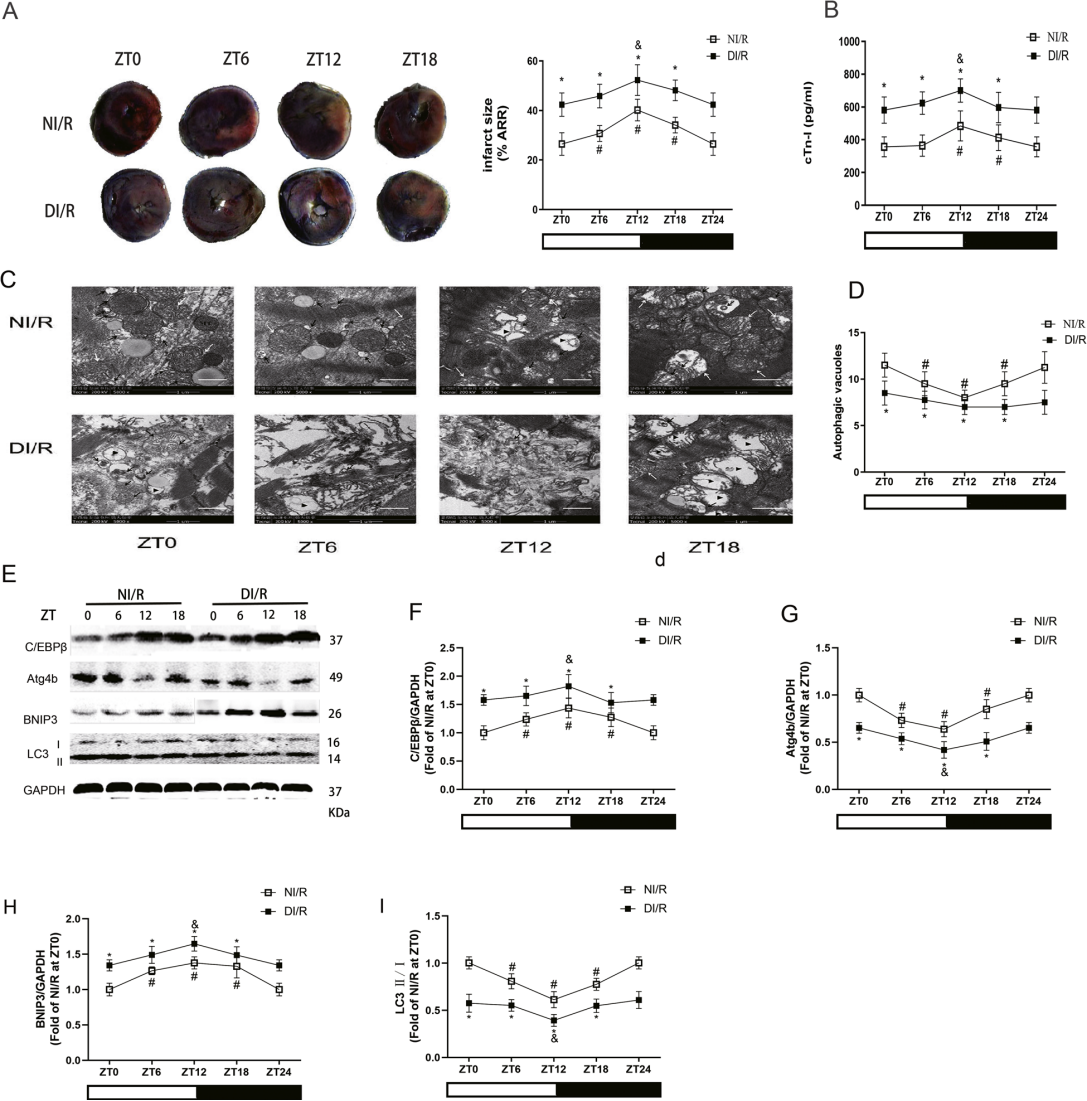
The circadian dependence of MI/RI in diabetic rats was attenuated, which was associated with disordered rhythm of mitophagy. **A** Infarct size was detected by TTC. Scale bar: 2 mm. **B** The serum level of cTn-I was detected by ELISA in non-diabetes or diabetes with I/R insult. **C** and **D** The ultrastructural changes and autophagic vacuoles of rat hearts were detected by TEM (MT: normal mitochondrion, white arrows: swollen mitochondrion, ▶: disorganized and vacuolated mitochondrion, black arrows: autophagosome/autophagic vacuole, Scale bar: 1 μm. **E**–**I** The protein levels of C/EBPβ (**F**), Atg4b (**G**), BNIP3 (**H**) and LC3 II/I (**I**) were detected by western blotting in the myocardial tissues of non-diabetes or diabetes with I/R insult. *n* = 6 per group. ^*^P < 0.05 versus NI/R within ZT; ^#^*P* < 0.05 versus ZT0 within NI/R; ^&^*P* < 0.05 versus ZT0 within DI/R.

We next determined the activity and rhythm of mitophagy in diabetic and non-diabetic rats after MI/RI. We found that the number of autophagosomes and autolysosomes showed rough oscillations, with a minimum at ZT12 in non-diabetic MI/RI rats (Fig. 2C, D). Compared with the non-diabetic rats, the mitochondrial damage was more serious in diabetic rats and the number of autophagosomes was significantly reduced, with no rhythmic fluctuations at different times. In accordance with the changes in autophagosomes and autolysosomes, the expression of mitophagy genes C/EBPβ (Fig. 2F) and BNIP3 (Fig. 2H) peaked at ZT12, and Atg4b (Fig. 2G) and LC3 II/I (Fig. 2I) expression reached a minimum at ZT12, with a circadian clock-dependent rhythm in the non-diabetic rats. However, the expression of these proteins showed disordered rhythm in diabetic rats. On the other hand, compared with NI/R group at the same time point, the protein levels of C/EBPβ and BNIP3 were significantly increased in DI/R group and the levels of Atg4b and LC3 II/I were significantly decreased. These results suggest that the circadian mitophagy rhythm is a vital factor in myocardial mitochondrial function, and that mitochondrial damage and mitophagy rhythm disorder may be the mechanism of increased vulnerability to MI/RI in diabetes.

### The increased vulnerability to MI/RI in diabetes is associated with HDAC3-mediated disruption of circadian gene expression oscillations by inhibiting mitophagy

**Fig. 3:**
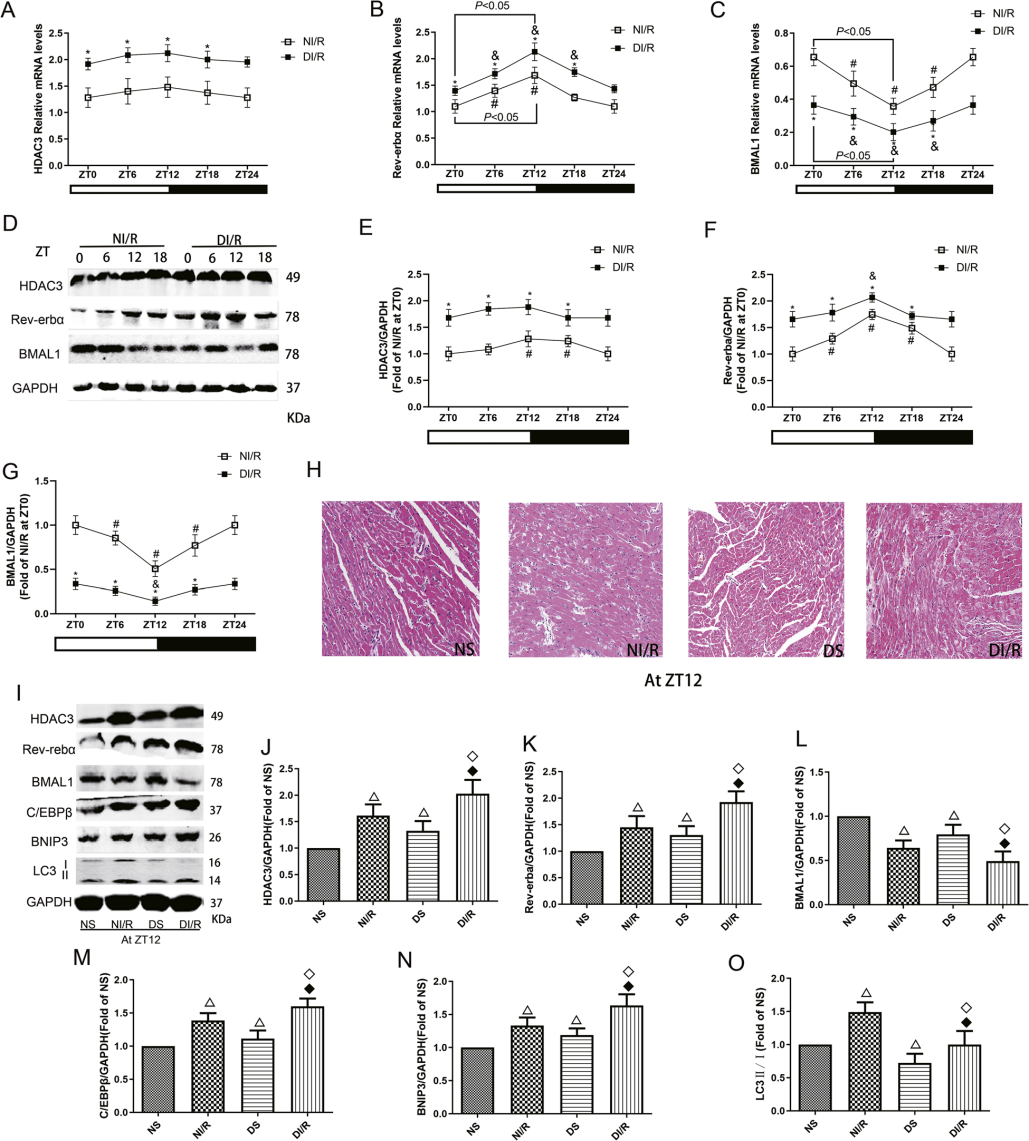
The increased vulnerability to MI/RI in diabetes is associated with HDAC3 mediated disruption of circadian gene expression oscillations by inhibiting mitophagy. **A**–**C** The mRNA levels of HDAC3 (**A**), Rev-erbα (**B**), and BMAL1 (**C**) were analysed by qPCR. **D**–**G** The protein levels of HDAC3 (**E**), Rev-erbα (**F**), and BMAL1 (**G**) were analysed by western blotting in myocardial tissues of non-diabetic and diabetic rats with I/R insult. **H** HE staining of hearts from NS, NI/R, DS, or DI/R mice at ZT12. Scale bar: 100 μm. **I**–**O** The protein levels of HDAC3 (**J**), Rev-erbα (**K**), BMAL1 (**L**), C/EBPβ (**M**), BNIP3 (**N**) and LC3 II/I (**O**) in NS, NI/R, DS or DI/R group at ZT12 were detected by western blotting. *n* = 6 per group. ^*^P < 0.05 versus NI/R within ZT; ^#^*P* < 0.05 versus ZT0 within NI/R; ^&^*P* < 0.05 versus ZT0 within DI/R; ^△^*P* < 0.05 versus NS; ^◇^*P* < 0.05 versus DS; ^◆^*P* < 0.05 versus NI/R.

The mRNA and protein expression of HDAC3 was not significantly rhythmic after I/R stimulation in non-diabetic or diabetic rats; at the same time point, the expression was significantly increased in the DI/R group compared with the NI/R group (Fig. 3A, E). Rev-erbα mRNA and protein levels showed different oscillation amplitudes in both non-diabetic and diabetic rats after I/R insult, and the amplitude in diabetic MI/R rats was significantly decreased and dislocated compared to that in non-diabetic MI/R rats (Fig. 3B, F). In the NI/R groups, the level of Rev-erbα protein peaked at ZT12; the protein level of Rev-erbα in DI/R groups was significantly increased than that in NI/R groups at same time. Under these conditions, the mRNA and protein expression of BMAL1 showed different amplitudes of oscillation in non-diabetic and diabetic rats subjected to MI/R insult, and the amplitude in diabetic rats was significantly decreased and dislocated compared to that in non-diabetic rats (Fig. 3C). BMAL1 protein expression showed the lowest level at ZT12 in the NI/R groups; the protein level of BMAL1 in DI/R groups was significantly lower than that in NI/R groups at same time.

We next investigated whether HDAC3-regulated circadian genes mediate mitophagy activation and are involved in the more serious MI/RI of diabetic rats at ZT12 time point, which is the time point with the most severe MI/RI. As shown in HE results, compared with the non-diabetic rats, the diabetic rats shown myocardium structural damage; after I/R insult, the myocardium showed myocardial fibrosis and oedema; and the myocardial tissue necrosis and inflammation in the D/IR group were more severe (Fig. 3H). Further, compared with the NS group, the protein levels of HDAC3 and Rev-erbα were increased in the DS group, accompanied with decreased BMAL1 levels (Fig. 3J–L). After I/R stimulation, the protein levels of HDAC3 and Rev-erbα were significantly increased in the DI/R group compared with the NI/R group, with further decreased BMAL1 levels. Moreover, the expression of autophagy-associated proteins C/EBPβ, BNIP3, and LC3 II/I was increased after I/R in the non-diabetes and diabetes groups compared with sham-operated groups (Fig. 3M–O). In addition, the level of LC3 II/I in diabetic rats was prominently decreased than those in non-diabetic rats, and after I/R insult, the degree of increase of these autophagy proteins was significantly smaller in diabetic rats than in non-diabetic rats.

### Genetic knockdown of HDAC3 recovered mitophagy status through the Rev-erbα/BMAL1 circadian pathway and significantly attenuated diabetic MI/RI

**Fig. 4:**
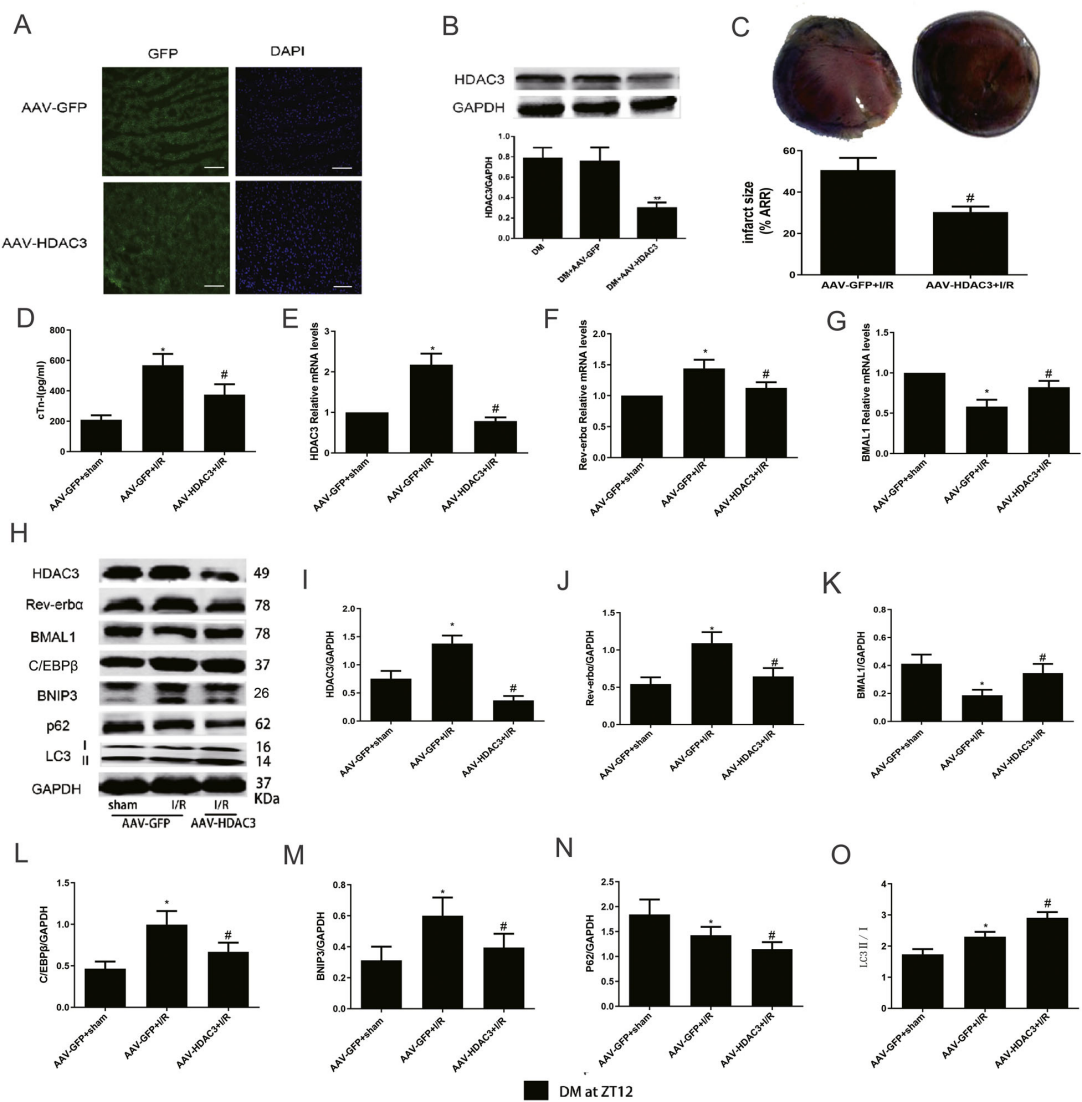
Genetic knockdown of HDAC3 recovered mitophagy status through the Rev-erbα/BMAL1 circadian pathway and significantly attenuated diabetic MI/RI. **A** Representative images of myocardial tissue GFP and DAPI was detected by immunofluorescence staining after AAV-GFP and AAV-HDAC3 transfection, scale bar = 100 μm. **B** Protein expression of HDAC3 was analysed by western blotting. **C** Infarct size was measured by TTC. **D** The serum level of cTn-I (**D**) was measured by ELISA assay kits. **E**–**G** The mRNA expression of HDAC3 (**E**), Rev-erbα (**F**), and BMAL1 (**G**) were analysed by qPCR. **H**–**O** The protein levels of HDAC3 (**I**), Rev-erbα (**J**), BMAL1 (**K**), C/EBPβ (**L**), BNIP3 (**M**), P62 (**N**), and LC3 II/I (**O**) were measured by western blotting. *n* = 6 per group. ^**^P < 0.01 versus DM; ^*^P < 0.05 versus AAV-GFP+sham; ^#^*P* < 0.05 versus AAV-GFP^+^I/R.

Next, we determined the mechanism by which hyperglycaemia-induced HDAC3 upregulation aggravated MI/RI in diabetic rats. Three weeks after HBAAV9-r-HDAC3 shRNA1-GFP and HBAAV9-GFP NC injection, we found that the GFP signals were clearly visible, indicating successful transfection and expression of AAV-GFP-NC and AAV-GFP-HDAC3 (Fig. 4A). Compared with the DM control and AAV-GFP transfection, AAV-HDAC3 transfection substantially decreased HDAC3 protein levels (Fig. 4B). Downregulation of HDAC3 significantly reduced the infarct size compared to that of the AAV-GFP-NC group after I/R (Fig. 4C). Furthermore, the serum level of cTn-I (Fig. 4D) was also obviously blunted in the AAV-HDAC3 group.

To explore the role of HDAC3 expression knockdown in circadian gene and autophagy in MI/RI in diabetic rats, we next investigated the expression of circadian genes and autophagy-related genes. The mRNA and protein levels of HDAC3 (Fig. 4E) in the I/R group were obviously increased than that in sham group of diabetic rats, with the same result for Rev-erbα mRNA and protein levels (Fig. 4F, J). However, the tendency was reversed notably by HDAC3 knockdown. The mRNA and protein levels of BMAL1 (Fig. 4G, K) were significantly increased in the AAV-HDAC3+I/R group compared with the AAV-GFP+I/R group. The expression of C/EBPβ (Fig. 4L), BNIP3 (Fig. 4M), and LC3 II/I (Fig. 4O) in diabetic MI/R rats were significantly increased compared with those of sham group, with decreased P62 expression (Fig. 4N). AAV-HDAC3 transfection showed obviously increased LC3 II/I levels and decreased P62 expression. Moreover, compared with AAV-GFP+I/R group, AAV-HDAC3 transfection significantly reversed the increased protein levels of C/EBPβ and BNIP3.

### High glucose aggravated H/R injury by reducing autophagic flow levels and mitophagosome with mitochondrial dysfunction in neonatal rat cardiomyocytes

**Fig. 5:**
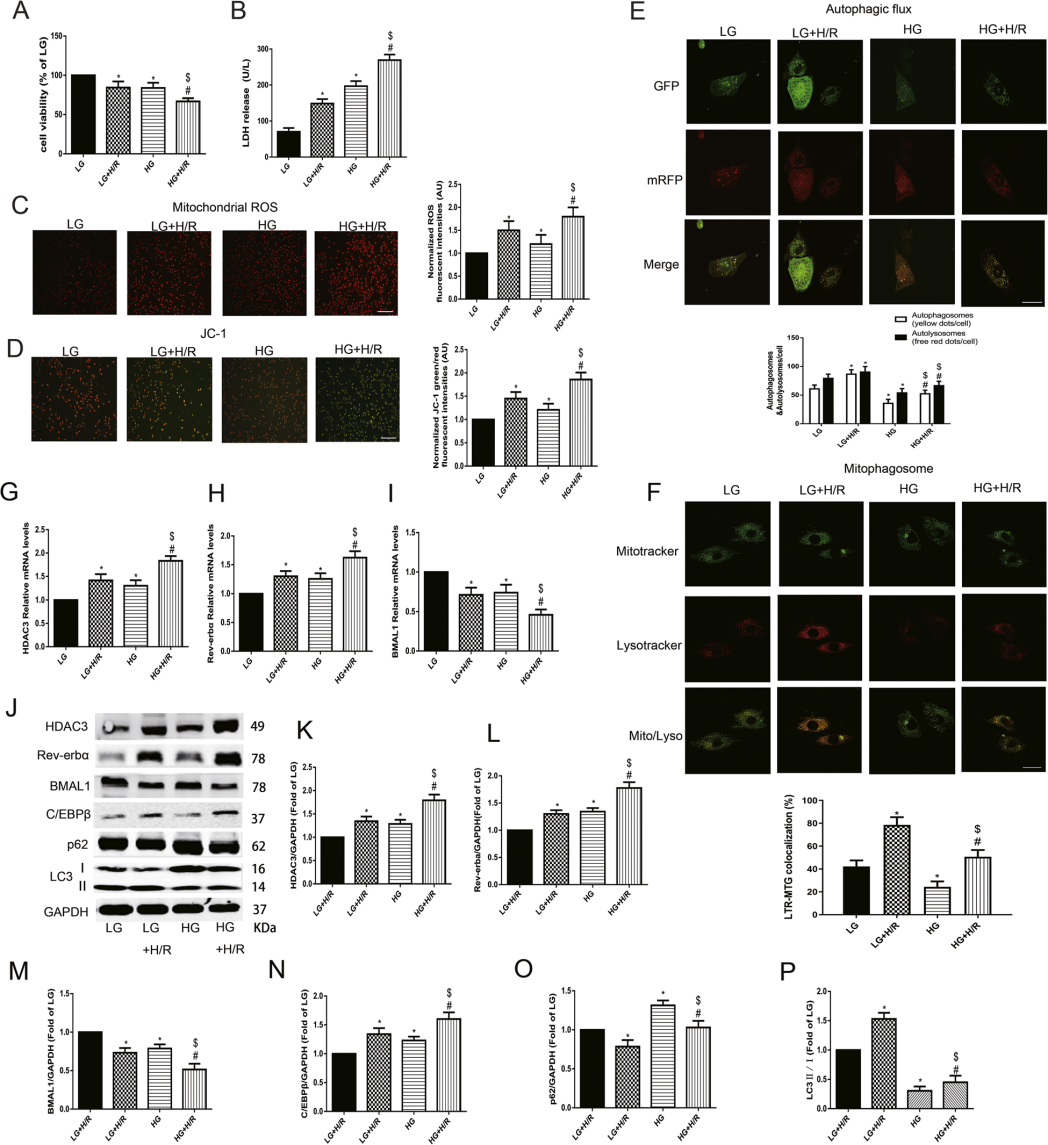
Upregulated expression of HDAC3 mediated Rev-erbα/BMAL1 in high glucose to increase the vulnerability to H/R by inhibiting mitophagy in neonatal rat cardiomyocytes. **A** Cell viability was analysed by CCK-8 kit. **B** Serum level of LDH was detected by ELISA kit. **C** and **D** Mitochondrial ROS (**C**) and JC-1 (**D**) were detected to analyse mitochondrial function. Scale bar: 100 μm. **E** Tandem fluorescent mRFP-GFP-LC3 adenovirus was used to detect the autophagic flux. Scale bar: 20 μm. **F** MitoTracker Green and LysoTracker Red staining were used to detect mitophagosome formation. Scale bar: 20 μm. **G**–**I** The mRNA levels of HDAC3 (**G**), Rev-erbα (**H**) and BMAL1 (**I**) were analysed by qPCR. **J**–**P** The protein levels of HDAC3 (**K**), Rev-erbα (**L**), BMAL1 (**M**), C/EBPβ (**N**), P62 (**O**) and LC3 II/I (**P**) were analysed by western blotting in the cultured neonatal rat cardiomyocytes. *n* = 6 per group. ^*^P < 0.05 LG; ^#^*P* < 0.05 versus HG; ^$^*P* < 0.05 versus LG+H/R.

HG significantly decreased cell viability, which was further aggravated by H/R stimulation (Fig. 5A). The level of LDH release (Fig. 5B) in neonatal rat cardiomyocytes was increased under HG conditions and further increased by H/R stimulation. Moreover, HG and H/R stimulation significantly increased the mitochondrial ROS production and JC-1 monomer/aggregate ratio (Fig. 5C, D). H/R insult significantly increased the number of autophagosome and autolysosome puncta with increased mitophagosome formation under both LG and HG conditions (Fig. 5E, F). However, HG decreased the levels of autophagic flux and mitophagosome than that in LG group. In HG+H/R group, the autophagic flux and mitophagosome were increased than that in HG group, but the increased level was obviously lower than that in LG+H/R group. These results indicate that under low-glucose conditions, H/R treatment can significantly increase the level of mitophagy; while under high-glucose conditions, the basal level of mitophagy is downregulated, and the increase in the amplitude of the mitophagy level after H/R insult is significantly reduced.

### HG-induced HDAC3 activation blunted Rev-erbα/BMAL1 pathway and decreased mitophagy status, which resulted in the increased vulnerability to H/R in neonatal cardiomyocytes

To evaluate the roles of HDAC3-orchestrated circadian clock gene oscillations and mitophagy in cardiomyocytes under HG and H/R insult, we then detected the mRNA and protein expression of related genes in neonatal cardiomyocytes. HG increased the mRNA levels of HDAC3 (Fig. 5G) and Rev-erbα (Fig. 5H), and decreased the mRNA level of BMAL1 (Fig. 5I). These changes were further increased by H/R. HDAC3 (Fig. 5K) and Rev-erbα (Fig. 5L) protein levels higher in the HG+H/R group than the LG+H/R group, with decreased level of BMAL1 (Fig. 5M). However, compared with those in the LG group, decreased autophagy activation levels were detected in the HG group, indicated by the decreased LC3 II/I ratio (Fig. 5P) and increased C/EPBβ (Fig. 5N) and P62 (Fig. 5O) expression. H/R stimulation increased autophagy activation under LG or HG conditions. However, the level of LC3 II/I was decreased and C/EPBβ expression was increased in the HG+H/R group than in the LG+H/R group (Fig. 5N, P). All these results demonstrated that high expression of HDAC3 under HG conditions influenced circadian clock genes to inhibit the activation of mitophagy, and ultimately aggravated H/R injury.

### Knockdown of HDAC3/Rev-erbα expression improved BMAL1 expression and mitophagy status, and ultimately attenuated H/R injury in neonatal rat cardiomyocytes exposed to HG

**Fig. 6:**
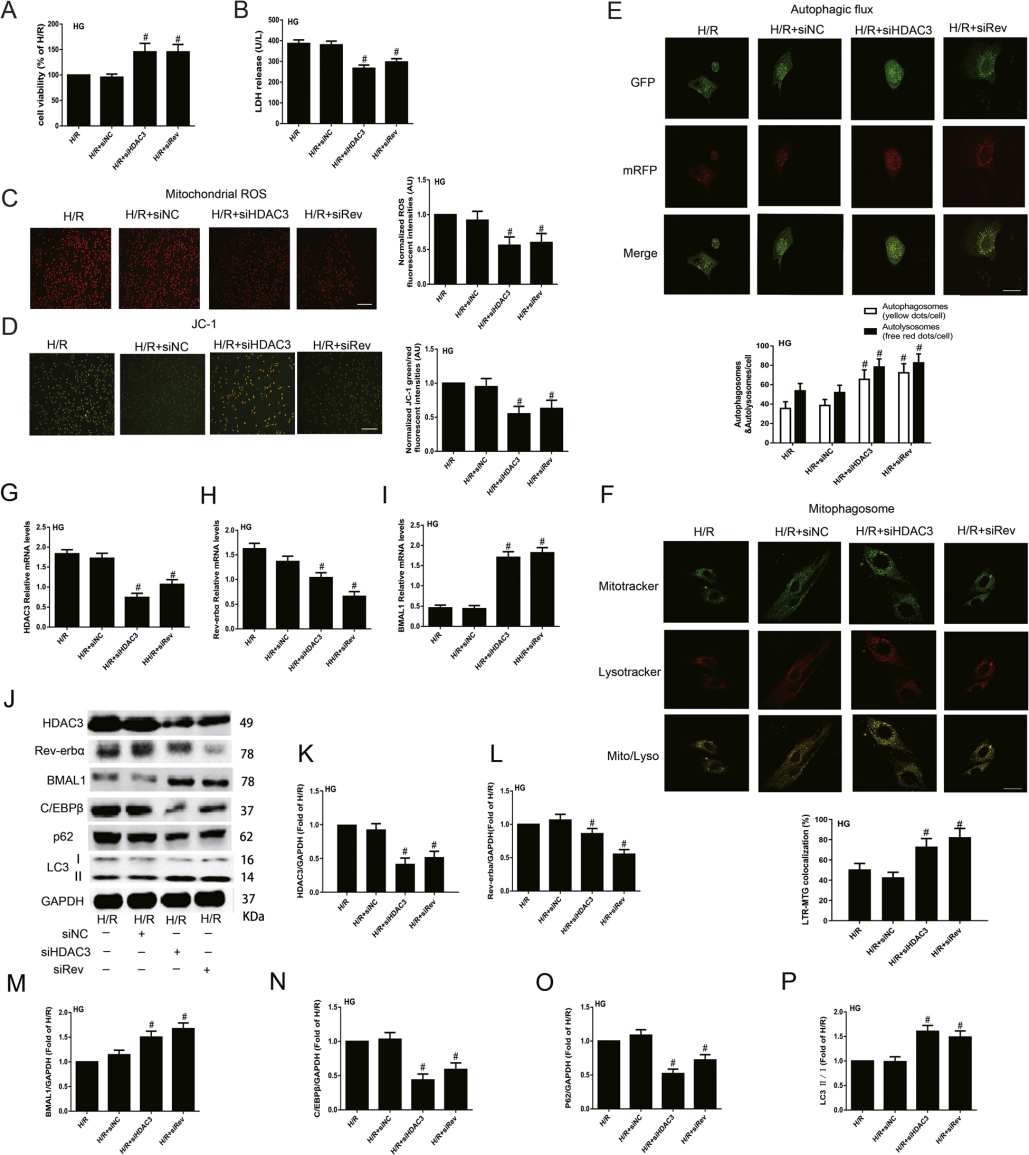
Knockdown of HDAC3/Rev-erbα expression attenuated neonatal rat cardiomyocytes H/R injury by upregulating BMAL1 expression to activate mitophagy under HG condition. **A** Cell viability was analysed by CCK-8 kit. **B** The serum level of LDH was analysed by ELISA kit. **C** and **D** Mitochondrial ROS (**C**) and JC-1 (**D**) were detected to analyse mitochondrial function. Scale bar: 100 μm. **E** Tandem fluorescent mRFP-GFP-LC3 adenovirus was used to detect autophagic flux. Scale bar: 20 μm. **F** MitoTracker Green and LysoTracker Red staining were used to detect mitophagosome formation. Scale bar: 20 μm. **G**–**I** The mRNA levels of HDAC3 (**G**), Rev-erbα (**H**), and BMAL1 (**I**) were analysed by qPCR. **J**–**P** The protein levels of HDAC3 (**K**), Rev-erbα (**L**), BMAL1 (**M**), C/EBPβ (**N**), P62 (**O**), and LC3 II/I (**P**) were analysed by western blotting in the cultured neonatal rat cardiomyocytes. *n* = 6 per group. ^#^*P* < 0.05 versus H/R.

To explore the mechanisms of HDAC3 in circadian gene-regulated mitophagy in HG+H/R-induced injury of neonatal rat cardiomyocytes, we used siRNA to knock down the expression of HDAC3 or Rev-erbα under HG conditions. We found that HDAC3-siRNA and Rev-siRNA obviously decreased H/R injury, detected by increased cell viability and decreased LDH release and mitochondrial ROS production (Fig. 6A–C). The MMP was significantly increased by siHDAC3 and siRev in cultured neonatal cardiomyocytes exposed to HG plus H/R insult (Fig. 6D). Autophagic flux was significantly increased in the siHDAC3 and siRev groups with increased mitophagosome formation compared with that in H/R group (Fig. 6E, F).

Compared with HG+H/R group, the mRNA and protein levels of HDAC3 (Fig. 6G, K) in the siHDAC3 and siRev groups were obviously decreased, with similar changes in Rev-erbα mRNA and protein levels (Fig. 6H, L). In addition, compared with those in the HG+H/R group, the mRNA and protein levels of BMAL1 were significantly increased (Fig. 6I, M) in the H/R+siHDAC3 and H/R+siRev groups. The expression of LC3 II/I was obviously increased after siHDAC3 and siRev infection compared with HG+H/R, accompanied with decreased C/EBPβ and P62 expression levels (Fig. 6N–P).

## Discussion

A variety of human and laboratory animal studies have shown that circadian clocks regulate biological cardiovascular rhythms in both health and disease, and this process also plays a significant role in myocardial ischaemic disease^10–12^. Our previous studies have confirmed that mitophagy is impaired during type 1 diabetic MI/RI, and Rap can partially restore the mitophagy level and rhythm of cardiomyocytes, thereby reducing MI/RI^6,20^. In this study, we first explored the roles of HDAC3-orchestrated circadian clock gene oscillations in mediating mitophagy in diabetic MI/RI and neonatal rat cardiomyocyte H/R injury under HG conditions. The novel findings of this study were as follows. First, circadian clock gene rhythmicity was attenuated in the hearts of diabetic rats. Second, diurnal variations were initiated in myocardial infarction and mitophagy after MI/RI in non-diabetic rats, and there was more severe injury in diabetic MI/R rats without diurnal variations, with impaired and rhythm-disordered mitophagy. Third, upregulated HDAC3 in diabetic rats mediates circadian gene Rev-erbα/BMAL1 oscillations disrupted, which impaired mitochondrial function and induced mitophagy dysfunction to increase vulnerability to MI/RI. Finally, cardiac-specific HDAC3 knockdown restored diabetes-induced vulnerability to MI/RI, and silencing of HDAC3 showed beneficial effects against diabetic MI/RI. Our present study conclusively shows that HDAC3-orchestrated circadian clock gene oscillations is a novel endogenous mechanism in diabetic MI/RI.

In recent years, an increasing number of studies have shown that clock gene oscillations play a significant role in heart disease; the myocardial infarct size caused by I/R at different times of day are related to the circadian clock^11–13^. Our results indicated that the myocardial infarct size and the degree of injury caused by MI/R in non-diabetic rats were time-dependent, with the greatest injury at ZT12, which is consistent with previous studies^11^. ZT12 corresponds to the sleep-to-wake period of nocturnal rodents. Therefore, the diurnal oscillations and responsiveness to stimulation (i.e., ischaemia) are in phase. These findings are similar to previous reports about cardiovascular effects of the circadian clock in humans, such as the onset of myocardial infarction or sudden cardiac death^26^. Genetic modulation of circadian clock timing, resulting in subtle circadian dyssynchronization, accelerated cardiac and renal disease, which is rescued by light/dark cycle-mediated circadian resynchronization^27^. Preclinical observations^11,12^ and our findings may have implications for the development of interventions aimed at reducing myocardial injury in clinical trials, demonstrating that the timing of coronary occlusion will affect the extent of subsequent infarction.

Clinical and experimental studies have shown that compared to individuals without diabetes, individuals with diabetes show increased vulnerability to MI/RI and resistance to various therapeutic methods^28,29^. Tolerance to ischaemia may exhibit variability associated with circadian rhythms^30^. The disorder and disruption of the circadian rhythm play a key role in the development of metabolic diseases such as diabetes, atherosclerotic plaque formation, and ischaemic heart disease^31^. The loss of synchronization between the clock and its environment in diabetic hearts may affect the development of diabetes^9^. As previously reported, we established type 1 diabetic animal models by intraperitoneal injection of STZ. After 8 weeks, we found that compared with those of non-diabetic rats, the circadian rhythm gene oscillations of diabetic rats are attenuated in the heart, as manifested by the alteration and attenuated amplitude of the clock output gene Rev-erbα and BMAL1 rhythmicity in STZ-induced diabetes, which is consistent with the results of Young et al.^9^. We hypothesize that these observations are due to changes in the circadian rhythms of zeitgebers in diabetes, which are consistent with a recently published study^32^. In further studies, we found that the heart of diabetic MI/RI rats at different time points had attenuation pattern of the clock gene, showing no significant fluctuations and more serious injury compared with non-diabetic rats.

As a member of the class I HDAC family, HDAC3 is a pivotal element in the circadian rhythm negative feedback loop and ischaemia heart disease^32,33^. HDAC3 activates Rev-erbα which promotes transcriptional repression to regulate the circadian rhythm. Rev-erbα directly binds to the BMAL1 gene promoter through two retinoid-related orphan receptor binding sites and inhibits its activity^34^. BMAL1 is an important regulatory mechanism in the circadian rhythm of human energy metabolism. BMAL1 knockout mice show circadian rhythm disorder, β cell secretion disorder, hyperglycaemia, and impaired glucose tolerance and ultimately develop diabetes^35,36^. Recent studies have indicated that the degree of myocardial injury, cardiac function changes, and myocardial remodelling after AMI are closely related to the circadian rhythm of the environment in which they are located; the oscillating amplitude of the circadian clock genes (Rev-erbα, BMAL1) in the ischaemic region exacerbates the development of myocardial infarction and the occurrence of heart failure, suggesting that myocardial damage is related to the circadian rhythm regulated by circadian clock genes^11,37,38^. In our study, after MI/R insult, there were diurnal variations of injury in non-diabetic rats. There was aggravated injury and attenuated diurnal variation in diabetic rats compared with non-diabetic rats, consistent with increased expression of HDAC3 and Rev-erbα and lower levels of BMAL1, which showed disordered rhythms. Furthermore, inhibition of HDAC3 expression by AAV9-HDAC3 protected against diabetic MI/RI by lowering the expression of Rev-erbα to upregulate BMAL1. These findings emphasized that HDAC3 orchestrated the alterations of circadian clock genes Rev-erbα and BMAL1 in the heart, probably because the changes in circulating zeitgebers during diabetes cause a loss of synchronization of stimulus-response coupling and play a role in aggravating MI/RI in diabetes.

Mitophagy is a key endogenous protection mechanism that maintains the integrity of the entire mitochondrial network, oxidative balance, and cell survival. Increased oxidative stress induced by hyperglycaemia and hyperlipidaemia under diabetic conditions can lead to mitophagy dysfunction^39^. Mitophagy is impaired during MI/R, and our previous studies have confirmed that mitophagy is more damaged in type 1 diabetic rats after MI/RI^6,26^. Studies have shown that autophagy activation shows strong circadian rhythms in several tissues, including the liver, heart, and skeletal muscle^22^. Myocardial mitophagy rhythm disorder is an important pathophysiological feature of MI/RI, and the autophagy agonist Rap partially restores the mitophagy level and rhythm of cardiomyocytes, thereby reducing the degree of MI/RI^6,40,41^. Here, we found that the level of mitophagy displays robust oscillations and minimums at ZT12 in non-diabetic rat hearts. Mitophagy level in diabetic rats was significantly reduced, with no rhythmic fluctuation. Moreover, I/R insult could significantly increase the mitophagy level in non-diabetic or diabetic rats. Mitochondrial damage is more serious in diabetic rats after MI/RI, and the increase degree of mitophagy was significantly lower in diabetic rats than in non-diabetic rats after MI/RI. These results indicate that mitophagy shows a significant rhythm in non-diabetic rats and is activated rhythmically after I/R insult to alleviate myocardial injury. In type 1 diabetes, the basal level of mitophagy is downregulated and is insufficiently increased after I/R stimulation, thus participating in diabetic MI/R vulnerability.

As a transcription factor, C/EBPβ is engaged in a variety of physiologic and pathophysiologic processes, and controls autophagy gene programmes and regulates mitophagy activity^41^. The level of C/EBPβ is highly rhythmic and the maintenance of mitophagy rhythm depends on the regulation of the core clock gene BMAL1 and is associated with the activation of C/EBPβ^23,24^. Therefore, the new important feature of mitophagy is its rhythm and relation to clock gene oscillation. In our present study, we found that the expression of C/EBPβ in non-diabetic rat myocardial tissue has circadian rhythm and that this rhythm is disturbed in diabetic rats with elevated levels. In a further study, C/EBPβ expression was upregulated in a time-dependent manner after MI/RI in non-diabetic rats and was further upregulated with oscillation imbalance in diabetic rats after MI/RI. When HDAC3 expression was downregulated, the level of C/EBPβ significantly decreased, with attenuated diabetic MI/RI. These results demonstrated that C/EBPβ, as a circadian rhythm gene, plays a vital role in diabetic MI/RI, possibly by regulating mitochondrial function and mitophagy.

BNIP3 is a key signalling factor involved in mitochondrial dysfunction, mitophagy, and cardiomyocyte death, which can be induced during ischaemia or hypoxia stress^42^. BNIP3 localized in mitochondria interacts with autophagosome-localized LC3, acting as an LC3-binding receptor on mitochondria, and mainly activates excessive mitophagy, causing cell death^43,44^. BNIP3 deficiency significantly reduced neuronal mitophagy and apoptosis^45^. Previous studies have suggested that BNIP3 is an important element that regulates MI/RI and cardiac remodelling^46–48^. In our present study, we found that the expression of BNIP3 was obviously upregulated in diabetic rats compared with non-diabetic rats and was further upregulated after I/R with aggravated injury, in accordance with increased LC3 II/I level in non-diabetic rats after MI/R stimulation and downregulated LC3 II/I level in diabetic rats with aggravated injury. When HDAC3 expression was downregulated, the level of BNIP3 significantly decreased, with obviously increased LC3 II/I expression and mitophagy levels that attenuated diabetic MI/RI. These results demonstrated that BNIP3 plays a vital role in diabetic MI/RI by inducing mitochondrial dysfunction and lowering mitophagy.

In conclusion, our present study demonstrated that HDAC3 is a novel mechanism in diabetic MI/RI by regulating circadian gene oscillations to induce mitophagy dysfunction, and knockdown of HDAC3 expression can relieve diabetic MI/RI. HDAC3-orchestrated circadian clock gene oscillations may provide an effective molecular target for the prophylaxis and treatment of diabetic MI/RI.

## Supplementary information

Supplementary table
