# Supplementary material for: Roles of HDAC3-orchestrated circadian clock gene oscillations in diabetic rats following myocardial ischaemia/reperfusion injury
Source: Cell Death Dis. 2021 Jan 7;12(1):43. doi: 10.1038/s41419-020-03295-y (PMC7791027; doi:10.1038/s41419-020-03295-y)
Supplement: Supplementary file 1 — Supplementary table [file 41419_2020_3295_MOESM1_ESM.docx]

**Supplementary**

**Table 1 Comparison of basic conditions of non-diabetic rats and diabetic rats after 8 weeks**

|  | N group | D group |
| --- | --- | --- |
| Water intake (ml/kg/d) | 238.4±8.7 | 712.0±37.6^*^ |
| Food intake (g/kg/d) | 72.3±5.6 | 208.6±13.0^*^ |
| Weight (g) | 389.2±21.8 | 185.6±16.4^*^ |
| blood glucose (mmol/L) | 4.8±1.5 | 27.6±7.0^*^ |

All data are expressed in $\overline{x}$±s, n=6 per group. ^*^*P*< 0.05 versus N group. N, non-diabetes; D, diabetes.
